# The association of clinical characteristics and liver stiffness measurement with liver viscosity measured by shear wave elastography in patient with non-alcoholic fatty liver disease

**DOI:** 10.2478/abm-2026-0006

**Published:** 2026-04-30

**Authors:** Anchasa Opasatian, Natthaporn Tanpowpong, Pisit Tangkijvanich

**Affiliations:** Department of Radiology, Faculty of Medicine, Chulalongkorn University, Bangkok 10330, Thailand; Center of Excellence in Hepatitis and Liver Cancer, Department of Biochemistry, Faculty of Medicine, Chulalongkorn University, Bangkok 10330, Thailand

**Keywords:** liver fibrosis, liver inflammation, magnetic resonance elastography, non-alcoholic fatty liver disease, viscosity plane-wave ultrasound

## Abstract

**Background:**

Viscosity plane-wave ultrasound (Vi.PLUS) measures tissue viscosity by using shear wave dispersion within tissues. The tissue viscosity is associated with the degree of liver necroinflammation, which is an important mechanism that leads to the development of liver fibrosis (LF).

**Objectives:**

This study aimed to examine the association of clinical characteristics and liver stiffness measurement (LSM) by magnetic resonance elastography (MRE) with liver viscosity measurement by shear wave elastography (SWE) in patients with non-alcoholic fatty liver disease.

**Methods:**

This monocentric cross-sectional study with retrospective data collection included 164 patients. The medical records of patients aged ≥18 years with non-alcoholic fatty liver disease (NAFLD) and who underwent MRE and Vi.PLUS at King Chulalongkorn Memorial Hospital were reviewed. The viscosity measurements were performed according to guidelines.

**Results:**

Of the 164 patients, 11 (6.7%) had invalid or unreliable measurements by elastographic technique and 4 (2.4%) were excluded due to incomplete clinical data. This resulted in 149 subjects being included in the final analysis. The study found good correlation between viscosity measurements by shear wave elastography and LSM by MRE (*r* = 0.74, *P* < 0.001). Multivariate regression analysis found that body mass index (*P* = 0.032) and LSM by MRE (*P* < 0.001) were independently associated with Vi.PLUS values.

**Conclusion:**

In conclusion, viscosity measured by shear wave elastography is a non-invasive method of assessing liver inflammation with good correlation to LF. This may aid the early diagnosis of liver inflammation in patients with NAFLD.

Non-alcoholic fatty liver disease (NAFLD) is one of the most common causes of chronic liver disorder with a prevalence of 25% worldwide [1]. This leads to the significant global disease burden of NAFLD [2].

Patients with NAFLD often have metabolic syndrome [3] due to both conditions having pathophysiological mechanisms of which insulin resistance is a major factor. Metabolic syndrome is defined as the presence of any 3 of the following 5 components: 1. abdominal obesity, defined as a waist circumference ≥90 cm (36 inches) in males and ≥80 cm (32 inches) in females or body mass index (BMI) >25 kg/m^2^ in the Asian population. BMI can be used in case the waist circumference is not obtainable; 2. serum triglycerides ≥150 mg/dL (1.7 mmol/L) or drug treatment for elevated triglycerides; 3. serum high-density lipoprotein (HDL) cholesterol <40 mg/dL (1 mmol/L) in males and <50 mg/dL (1.3 mmol/L) in females or drug treatment for low HDL cholesterol; 4. blood pressure ≥130/85 mmHg or drug treatment for elevated blood pressure; and 5. fasting plasma glucose (FPG) ≥100 mg/dL (5.6 mmol/L) or drug treatment for elevated blood glucose.

The range of diseases in NAFLD extends from simple steatosis to progressive steatohepatitis. The condition can advance to cirrhosis, leading to associated complications such as hepatocellular carcinoma [4]. Fibrosis of liver in patient with NAFLD is the most significant predictor of prognosis [5]. Thus, methods for assessing hepatic steatosis (HS), liver fibrosis (LF), and inflammation which are non-invasive, repeatable, and precise are of great clinical value. The gold standard for assessing LF is liver biopsy. However, liver biopsy is an invasive procedure with some limitations, including requirement for skilled practitioners, potential sampling errors, variability between operators and within individuals, high costs, and risk of complications. Complications of percutaneous liver biopsy are bleeding, organ perforation, and infection [6, 7].

Magnetic resonance imaging and ultrasonography have been implemented as a non-invasive tool for staging of LF. Liver stiffness measurement (LSM) obtained from magnetic resonance elastography (MRE) has been shown to accurately assess LF [8, 9]. However, the major drawbacks of MRE are high equipment costs, requires a specialist, low accessibility, and long examination length. Thus, ultrasound is more commonly performed in clinical practice. Ultrasound-based elastography techniques such as acoustic radiation force impulse (ARFI), produce shear waves through the application of a focused ultrasound beam's push pulse. The tissue elasticity or Young's modulus (*E*) is then calculated using the measured shear wave speed and Young's modulus. The formula for calculation is *E* = 3*pv*^2^, where *ρ* is the density of tissue in kg/m^3^ and *v* is the shear wave speed. The formula assumes that the tissue is entirely elastic, incompressible, exhibits a linear elastic response, and maintains a constant density of 1,000 kg/m^3^. Tissue elasticity is related to liver stiffness, which can be used in assessing LF [18]. Viscosity plane-wave ultrasound (Vi.PLUS) measures tissue viscosity by using shear wave dispersion within the tissues. Tissue viscosity is associated with the degree of liver necroinflammation [10], which plays a critical role in LF development.

Fat quantification techniques based on ultrasound attenuation rely on decrease in the energy of acoustic signals as they propagate through tissue. The decrease in signal return to the transducer due to this energy loss manifests as hypoechoic areas in deeper tissues. The presence of fat in the tissue amplifies attenuation, consequently prolonging the signal delay. Measuring this energy loss enables the quantification of tissue fat content. This technique allows assessment of HS [22].

Serum aminotransferases are clinically used as indicators of liver cell injury. Alanine aminotransferase (ALT, serum glutamic-pyruvic transaminase [SGPT]) and aspartate aminotransferase (AST, serum glutamic-oxaloacetic transaminase [SGOT]) are the most commonly measured serum aminotransferases. The normal range for AST is between 5 and 35 U/L and ALT between 0 and 40 U/L. Most liver diseases and disorders that involve the liver can result in elevated levels of AST and ALT. Examples of the aforementioned conditions are infections, NAFLD, drugs, acute and chronic heart failure, and carcinoma [11].

This study aimed to examine the association of the clinical characteristics and LSM by MRE with viscosity measurement by shear wave elastography (Vi.PLUS values) in patients with NAFLD.

## Methods

This monocentric cross-sectional study with retrospective data collection included 164 patients. The medical records of patients aged ≥18 years with NAFLD and who underwent MRE and Vi.PLUS between March 1, 2022 and June 30, 2022 at King Chulalongkorn Memorial Hospital were reviewed.

The eligibility criteria included age 18 years and above, NAFLD, fatty changes of the liver observed by abdominal ultrasound, obtainable serum AST, ALT levels from medical records, and underwent MRE and Vi.PLUS at King Chulalongkorn Memorial Hospital. Patients with increased consumption of alcohol (ethanol intake >210 g/week in male and >140 g/week in female), other chronic liver disease with known etiology (autoimmune hepatitis, hepatitis B virus infection, hepatitis C virus infection, primary sclerosing cholangitis or primary biliary cholangitis), ascites, obstruction of the hepatobiliary system, elevated ALT levels >5 times the upper normal limit, known history of malignancy, focal liver lesion, and heart failure resulting in hepatic congestion were excluded from the study.

Clinical data including biological sex, age at the time MRE was performed, weight, height, abdominal circumference, serum AST and ALT levels, and radiological data, including MRE report and Vi.PLUS report, were gathered from patients' medical records.

Approval was granted by the ethics committee of the Faculty of Medicine, Chulalongkorn University (COA no. 0178/2024).

### Viscosity measurement method

A C6-1X convex transducer with UltraFast™ software which is available on the Aixplorer Mach 30 ultrasound system was used for viscosity measurement.

**Figure 1.**
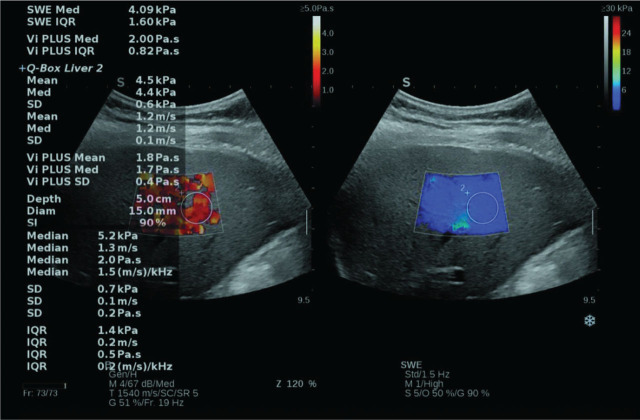
The image of a Vi.PLUS measurement, which was performed in a NAFLD patient. The viscosity is displayed in the left part of the image. Colors close to yellow white are indicative of high viscosity, while red means low viscosity. Numeric Vi.PLUS results (expressed in Pa.S) are shown on the left side of the image. NAFLD, non-alcoholic fatty liver disease; SD, standard deviation; SI, stability index; Vi.PLUS, Viscosity plane-wave ultrasound.

In accordance with The World Federation for Ultrasound in Medicine and Biology (WFUMB) and European Federation of Societies for Ultrasound in Medicine and Biology (EFSUMB) guidelines, the viscosity measurements were performed using the following protocols. Patients were examined in fasting conditions and using the intercostal approach in the right hepatic lobe. The position was supine with the right arm positioned above the head. The viscosity measurement box was placed at least 1.5 cm beneath the Glisson's capsule of the right lobe of the liver (**Figure 1**). Large vascular structures or bile ducts and rib shadows should not be included in the measurement box. Image acquisitions were performed during resting respiratory position (breath-hold without deep inspiration).

The Q-Box displays the mean, median, minimum, maximum, and standard deviation (SD) of the measurements, together with the depth, the diameter of the region of interest (ROI), Interquartile Range (IQR), and the Stability Index (SI).

### Statistical analysis

After reviewing the radiological and clinical reports, continuous data were analyzed by mean and SD while categorical data were analyzed by frequency and percentage. The statistical analysis was conducted using SPSS version 22.0. The association of BMI, abdominal circumference, serum AST and ALT levels, and LSM by MRE with viscosity measurement by shear wave elastography (SWE) was evaluated by using Pearson correlation and linear regression model.

## Results

### Baseline characteristics

The study enrolled 164 patients with NAFLD. Of the 164 patients, 11 (6.7%) had invalid or unreliable measurements by elastographic technique and 4 (2.4%) were excluded due to incomplete clinical data. This resulted in149 subjects being included in the final analysis. In accordance with the proposed guidelines for interpretation of liver stiffness by MRE [12], fibrosis distribution was as follows: 63.1% (94/149) of patients had normal liver stiffness, 18.1% (27/149) of patients had normal or inflammation of liver, 6.7% (10/149) of patients had stage 1–2 fibrosis, 1.3% (2/149) of patients had stage 2–3 fibrosis, 5.4% (8/149) of patients had stage 3–4 fibrosis, and 5.4% (8/149) of patients had stage 4 fibrosis **(Table 1)**.

**Table 1.** Displays the main characteristics of the patients and measurements

**Parameter (mean ± SD)**	**n = 149**
Age (years)	55.8 ± 13.6
Gender	
Males	78/149 (52.3%)
Females	71/149 (47.7%)
BMI (kg/m^2^)	27.7 ± 4.6
Abdominal circumference (cm)	96.2 ± 13.9
AST (U/L)	26.5 ± 11.7
ALT (U/L)	35.9 ± 23.5
Vi.PLUS (Pa.S)	2.0 ± 0.5
LSM by MRE (kPa)	2.6 ± 1.1
LF distribution by MRE	
Normal	63.1% (94/149)
Normal or inflammation	18.1% (27/149)
Stage 1–2 fibrosis	6.7% (10/149)
Stage 2–3 fibrosis	1.3% (2/149)
Stage 3–4 fibrosis	5.4% (8/149)
Stage 4 fibrosis	5.4% (8/149)

ALT, alanine aminotransferase; AST, aspartate aminotransferase; BMI, body mass index; LF, liver fibrosis; LSM, liver stiffness measurement; MRE, magnetic resonance elastography; SD, standard deviation; Vi.PLUS, viscosity plane-wave ultrasound.

### Association of clinical characteristics and LSM with viscosity measured by shear wave elastography (SWE)

**Figure 2.**
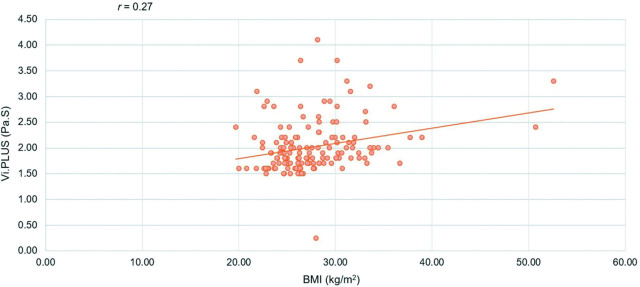
Relationship between BMI and Vi.PLUS; Pearson correlation coefficient, *r* = 0.27. BMI, body mass index; Vi.PLUS, Viscosity plane-wave ultrasound.

The correlations between the Vi.PLUS values and age, BMI, abdominal circumference, AST, ALT, and LSM by MRE were examined using univariate and multivariate statistical analyses. The univariate regression analysis showed an independent association between Vi.PLUS measurements and the following parameters: age (*P* = 0.036), BMI (*P* = 0.001), abdominal circumference (*P* < 0.001), AST (*P* < 0.001), and LSM by MRE (*P* < 0.001). However, ALT (*P* = 0.151) was not associated with Vi.PLUS values. Multivariate regression analysis found that BMI (*P* = 0.032) and LSM by MRE (*P* < 0.001) were independently associated with Vi.PLUS measurements (*r* = 0.27, *P* < 0.001) (**Figure 2**).

**Figure 3.**
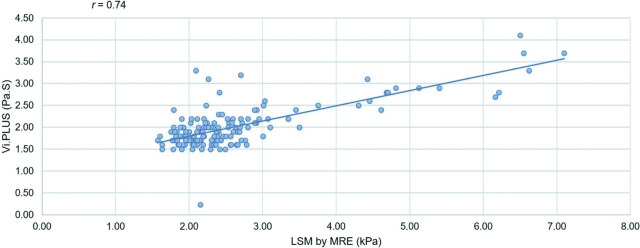
Relationship between LSM by MRE and Vi.PLUS; Pearson correlation coefficient, *r* = 0.74. LSM, liver stiffness measurement; MRE, magnetic resonance elastography; Vi.PLUS, Viscosity plane-wave ultrasound.

The study found good correlation between the Vi.PLUS values and LSM by MRE (*r* = 0.74, *P* < 0.001) (**Figure 3**).

## Discussion

NAFLD was first described in 1980. It is defined as the accumulation of hepatic fat, as evidenced by radiologic or histologic examination, in the absence of a coexisting etiology of chronic liver disease or secondary cause of steatosis [13] such as drugs and significant alcohol consumption. The histologic diagnosis of NAFLD is >5% steatotic hepatocytes in a liver tissue section [14].

Hepatic inflammation is an important mechanism that leads to the development of LF. Only a few studies incorporated non-invasive evaluation of tissue viscosity.

In their initial research, Deffieux et al. [20] conducted a study utilizing an ultrasound (US) imaging system to examine liver viscosity. Their findings suggested that liver stiffness proved to be a more reliable indicator for staging fibrosis; viscosity displayed a lesser predictive capability in this regard. Liver viscosity additionally demonstrated a modest ability to predict disease activity and levels of steatosis. Chen et al. [21] conducted a study assessing liver viscosity (Pa.s) alongside elasticity (kPa) using a US system in patients with different chronic liver conditions. Their findings indicated that viscosity exhibited lower predictive value in staging fibrosis compared with elasticity. In Sugimoto et al.'s [15] study, a multivariate analysis using histologic features as independent variables revealed significant associations. Specifically, fibrosis stage exhibited a significant correlation with shear wave (SW) speed (*P* = 0.037), while lobular inflammation grade showed a significant correlation with dispersion slope (*P* = 0.022). Thus, elasticity proved to be more effective than viscosity in predicting fibrosis stage, whereas viscosity demonstrated greater value in predicting the extent of necroinflammation.

Although viscosity is shown to be related to necroinflammation in histologic features, the association of viscosity with the AST and ALT values remains inconclusive. In a study conducted by Popa et al. [16] including only patients with NAFLD showed an independent association between Vi.PLUS values and LSM using 2D-SWE.PLUS (2D-ShearWave Elastography Plane-wave UltraSound), BMI, and abdominal circumference, whereas AST and ALT did not show independent associations with Vi.PLUS values. When multivariate regression analysis was done, only BMI and LSM by 2D-SWE.PLUS were associated with Vi.PLUS measurements. The study by Roxana et al. [17], which included a large cohort of patients with chronic liver diseases, showed that Vi PLUS measurements were independently associated with BMI, abdominal circumference, age, AST, and ALT in univariate regression analysis. In multiple regression analysis, abdominal circumference and AST and ALT values were associated with Vi.PLUS measurements. The 2 studies showed conflicting results and neither of them included the LSM measured by MRE.

MRE has been implemented in clinical practice for almost a decade, with widespread acceptance for both detecting and staging of LF. MRE enables direct visualization of shear waves propagation within the liver. An inversion algorithm embedded in the scanner automatically translates shear wave properties into an elastogram, or stiffness map, facilitating the calculation of liver stiffness. Currently, MRE is considered as the most accurate non-invasive diagnostic tool for both detecting and staging LF [19].

The univariate regression analysis in this study revealed an independent association between Vi.PLUS values and age, BMI, abdominal circumference, AST, and LSM by MRE. However, ALT did not exhibit an independent association with Vi.PLUS values. Multivariate regression analysis demonstrated that BMI and LSM by MRE were associated with Vi.PLUS measurements. Good correlation between Vi.PLUS measurements and LSM by MRE, which is related to LF, is observed in this study. While LF is the primary contributor to the elevated liver stiffness (measured by MRE) in chronic liver disease, it is essential to acknowledge that various pathological mechanisms can also elevate liver stiffness, potentially complicating the detection of fibrosis. These mechanisms comprise passive vascular congestion, inflammation, and portal hypertension [19]. The current standard MRE may not be able to differentiate various pathological mechanisms that can increase liver stiffness; however, the viscosity was found to be useful for predicting the degree of necroinflammation, which can also increase liver stiffness [15].

This study's limitation was the lack of a pathological report due to no patient who underwent liver biopsy. However, LSM by MRE has been shown to accurately assess LF [8, 9]. Low prevalence of patients with LF is also another limitation. Normal or inflammation of liver was found in 81.2% (121/149) of the patients. Only 28 patients (18.8%) had any degree of LF.

## Conclusion

In conclusion, the study found a good correlation between the Vi.PLUS values and LSM by MRE. Increase in BMI and liver stiffness were associated with high viscosity values. In the current clinical practice, US is a more accessible tool compared with MRE. The viscosity measured by shear wave elastography is a non-invasive method for assessing liver inflammation with good correlation to LF. This may aid the early diagnosis of liver inflammation and fibrosis staging and assist in guiding multidisciplinary treatment decision to provide the most optimized treatment for patients with NAFLD.
